# Vertebroplasty for acute painful osteoporotic fractures (VAPOUR): study protocol for a randomized controlled trial

**DOI:** 10.1186/s13063-015-0671-8

**Published:** 2015-04-12

**Authors:** William Clark, Paul Bird, Terrence Diamond, Peter Gonski

**Affiliations:** Department of Interventional Radiology, St George Private Hospital, Kogarah, NSW Australia; University of NSW, Sydney, NSW Australia

**Keywords:** Vertebroplasty, Osteoporotic, Fracture, Acute, Cement, Spinal, Pain, Interventional, Radiology

## Abstract

**Background:**

With increasing human longevity there is increasing prevalence of osteoporosis and of osteoporotic vertebral fractures. Most vertebral fractures do not require medical therapy for pain, but a minority are associated with severe pain and disability.

Vertebroplasty has been used increasingly for painful acute osteoporotic fractures. The best available evidence for vertebroplasty is provided by two placebo controlled trials which showed no significant clinical benefit of vertebroplasty over placebo. These were not acute fracture trials with the majority of fractures in both trials being well beyond the acute time frame of 6 weeks. There is evidence from an open label randomized controlled trial of vertebroplasty versus conservative therapy in acute fractures suggesting clinical efficacy in acute vertebral fractures.

**Methods:**

This is a blinded trial of Vertebroplasty for Acute Painful Osteoporotic fractURes - the VAPOUR trial. Patients greater than 60 years in age with new severe onset of back pain and osteoporotic vertebral fractures of less than 6 weeks duration will be enrolled. They will be randomized to receive either vertebroplasty or a placebo procedure. Data regarding pain, disability, and quality of life will be collected over a 6-month period. The enrolled patients and the outcome assessors will remain blinded for the duration of the trial.

**Discussion:**

The VAPOUR trial will apply similar methodology to the previous blinded trials but in a patient group with exclusively acute fractures and the most severe pain.

**Trial registration:**

ClinicalTrials.gov trial identifier: NCT01482793 registered on 28 November 2011.

## Background

With increasing human longevity there is increasing prevalence of osteoporosis. Vertebral body fractures are among the most common osteoporotic fractures. Whilst most cause minimal symptoms, a subset can cause severe acute fracture pain. Vertebroplasty has become a commonly used procedure for the management of painful osteoporotic vertebral fractures.

There is conflict in the best published evidence for vertebroplasty. There are two blinded randomized controlled trials (RCTs) published together in 2009 [1,2] which provide the highest quality evidence at present and show no benefit of vertebroplasty over placebo at any time point. Both enrolled similar patient groups, with osteoporotic fractures and back pain for up to 1 year. The average durations of back pain in these trials were 16 to 18 weeks in the larger trial [1] and 10 weeks in the smaller trial [2]. Neither trial found significant clinical advantage over placebo in the vertebroplasty group at any time point.

The largest open label RCT of vertebroplasty [3] limited enrolment to painful fractures less than 6 weeks duration. This trial compared vertebroplasty to usual care and found positively for vertebroplasty with enhanced reduction in pain score and also in the Roland Morris Disability score in the vertebroplasty group at each time point out to 12 months following the intervention. Similar findings were obtained in a large open label RCT of kyphoplasty versus conservative therapy [4] in a patient group with fracture pain less than 3 months duration.

A debate developed regarding the apparent discrepancy in findings between the blinded and the open label trials. Proponents of vertebroplasty argued that patient selection and procedural technique explained the negative findings in the blinded trials [5,6]. The Vertos II trial [3] was a trial of vertebroplasty for acute fractures whereas the two blinded trials [1,2] included predominantly non-acute fractures where the bone may have already healed. Patient selection could explain the difference. Technical considerations, and in particular the small volume of cement injected, were also questioned in the blinded trials [7]. Conversely, evidence-based medicine experts have argued that the design of the blinded trials was superior and that the apparent efficacy of vertebroplasty in the Vertos II trial was due to a placebo effect and the inherent bias in an open label trial [8-10].

A meta-analysis [11] of the two blinded RCTs attempted to answer the remaining question about the efficacy of vertebroplasty in the acute setting. The authors found 57 patients (25 vertebroplasty and 32 controls) from the combined enrolment of both blinded RCTs [1,2] to have had back pain for less than 6 weeks. This meta-analysis found no benefit of vertebroplasty over placebo at one week or one month. Analysis beyond one month was not presented. This meta-analysis contained little technical data with no reporting of the vertebral fracture levels, the inclusion of inpatients or not (inpatients were completely excluded in the larger trial and not reported in the smaller trial), and the cement volume injected (average 2.6 cc for both trials overall, but no reporting for this sub-group meta-analysis).

There is clearly a requirement for a resolution of the conflicting clinical evidence for vertebroplasty in the acute fracture setting. This requires a blinded RCT of vertebroplasty for acute vertebral fractures (defined as less than 6 weeks duration) with new onset of severe back pain (defined as pain score greater than 7/10). We are conducting such a trial: Vertebroplasty for Acute Painful Osteoporotic vertebral fractURes (The VAPOUR Trial).

### Mechanics of vertebroplasty

The mechanics of vertebroplasty underpin the treatment’s application and patient selection, and these principles need be clarified as they affect study design. We hypothesize that vertebroplasty is only effective when applied in the acute fracture setting in patients with severe pain. It can then potentially provide internal fixation and ameliorate the acute pain generated by fracture fragment motion. To achieve adequate internal fixation in a collapsing bone, we believe that the volume of injected cement is important to outcome and this volume will be measured. We also hypothesize that fractures in different parts of the spine are subject to different biomechanical forces which affect fracture behaviour. These segments may therefore respond differently to vertebroplasty. We will analyze the sub-groups of thoracic (T4-T10), thoraco-lumbar (T11-L2) and lumbar (L3-L5) as categorized in previous RCTs of spinal augmentation [3,4]. It is our anecdotal belief that the thoraco-lumbar segment is the region of the spine that responds best to vertebroplasty in the acute setting. This is the segment that is exposed to the largest flexion forces and accounts for the majority of “osteonecrotic” fracture clefts filled with either fluid or gas [12-14]. It has been previously demonstrated that thoracolumbar fractures are predisposed to “dynamic mobility” [15] which means that the vertebral body assumes different volumes in the erect and supine (or prone) positions. Such a fracture should benefit the most symptomatic relief from internal fracture fixation afforded by vertebroplasty by reducing fracture fragment motion.

The thoracic vertebrae (T4 to T10) undergo relatively reduced flexion force due to the bracing of the posterior elements by the rib cage. The reduced force across the fracture line in the thoracic segment may imply that natural healing is improved and that less mechanical advantage is obtained from vertebroplasty.

## Methods/design

### Trial design

The trial is a participant and outcome-assessor blinded randomized controlled trial of vertebroplasty for acute fractures. Acute fracture will be defined as new severe onset of back pain (of less than 6 weeks duration) associated with magnetic resonance imaging (MRI) or single-photon emission computed tomography (SPECT)-computed tomography (CT) evidence consistent with a recent fracture. MRI or SPECT-CT must be obtained within a week of patient enrolment. The MRI will include sagittal T1 weighted and fat suppressed T2 weighted images of the thoracic and lumbar spine. Deformity of the vertebral body associated with the high signal pattern (T2) and low signal pattern (T1) of acute fracture within the vertebral body will define recent fracture. Note that the precise duration of fracture is defined by careful questioning of the patient as to the date of onset of severe pain. The pain location should be consistent with the recent fracture identified on MRI. When MRI is contraindicated, then SPECT-CT will be obtained. Increased radioisotope uptake in the pattern of recent fracture associated with fracture deformity of the vertebral body will be accepted as evidence of recent fracture.

The trial is of essentially similar design as the previous two blinded RCTs [1,2]. It is a multicentre trial involving four centres in Sydney, Australia that have established vertebroplasty programs. Data will be collected at baseline, 3 days, 2 weeks, 1 month, 3 months and 6 months post intervention. The patient and the data collectors remain blinded for the duration of the trial.

### Patient selection

Patients referred for vertebroplasty will be screened for eligibility according to principle inclusion and exclusion criteria (Table 1). Those patients who are eligible for enrolment will be offered entrance into the trial. Vertebroplasty will not be available outside of the vertebroplasty trial, so that eligible patients are offered the choice between conservative care and enrolment in the trial. Ineligible patients will not be offered vertebroplasty. The conflicting evidence for the role of vertebroplasty in the management of acute osteoporotic fractures will be explained to the patient by the referring physician and the interventional radiologist. Informed consent to participate in the trial will be obtained prior to patient enrolment.

**Table 1:** **Principle inclusion and exclusion criteria**

**Inclusion criteria**	**Exclusion criteria**
Age >60	Inability to provide informed consent
Pain from one or two acute vertebral fractures	Chronic back pain requiring narcotic analgesia
MRI or SPECT-CT obtained within previous week	Presence of sciatica
Duration of fracture pain <6 weeks	Significant retropulsion into spinal canal
Pain >7/10 on numeric scale	Evidence of spinal malignancy
Patient can speak English	Active systemic infection

### Baseline assessment

After the patient has consented to inclusion in the trial, baseline medical history including general medical history, history of osteoporosis, previous fractures, previous spinal interventions and medications is recorded. Baseline measure of numeric pain scores and visual analogue pain scores are collected together with Roland Morris Disability score, QUALLEFFO quality of life, medication usage and EQ-5D. The baseline assessment is collected either on the day of the procedure or the day prior to the procedure, depending upon patient convenience.

### Random allocation

The subjects will be randomized to either the control intervention group or the vertebroplasty group using the method of minimization. This will be performed centrally at the National Health and Medical Research Council (NHMRC) Clinical Trials Centre, University of Sydney. The call to the NHMRC Clinical Trials Centre will be made once the patient is in the procedure room immediately prior to the procedure.

Randomization will be 1:1 to each treatment arm, and patients will be stratified by the following factors: * Age (<75, ≥75 years) * Degree of bone height loss (<50%, ≥50%) * Fracture due to trauma (Yes, No) * Patient taking steroids (Yes, No) * Hospital/site.

### Procedures

Patient will be laid prone on the procedure table and an oxygen mask applied. Pulse oximetry monitoring will be used continuously to monitor arterial oxygen saturation and pulse rate. Conscious sedation, comprising midazolam and fentanyl, will be administered intravenously.

Vertebroplasty will be performed according to the standard practice of the Interventional Radiology investigators in the trial. Local anesthetic will be injected into the soft tissues of the back and a 4-mm skin incision made to allow entrance of the vertebroplasty needle. An 11-gauge or 13-gauge vertebroplasty needle will be introduced into the vertebral body using a transpedicular approach and fluoroscopic imaging guidance. Either a unipedicular or bipedicular approach can be employed depending upon operator preference. AVAMAX (CareFusion Corporation) bone cement will be used for all vertebroplasty procedures. The cement is injected into the vertebral body using the approved kit technique during continuous fluoroscopic screening. The injection will be terminated when there is satisfactory distribution of the cement or if there is any cement leak into an adjacent structure. The proceduralists have been advised to attempt “maximal fill” of the vertebral body where possible. Ideally this means a cement distribution from the superior to the inferior end plate, from the medial cortex of the pedicle to the medial cortex of the contralateral pedicle and from the anterior cortex of the vertebral body to the posterior third of the vertebral body. Cement injection is always performed in the lateral screening projection with intermittent fluoroscopic checking in the anteroposterior (AP) projection. When these fluoroscopic end points are achieved in the AP and lateral projections, the injection is ceased. If there is significant venous, disc or spinal canal cement extrusion observed, then the injection is terminated. The vertebroplasty kits used in this trial include an injecting syringe with clear volumetric markings which facilitates volume measurement. The volume of cement injected in each vertebroplasty case will be recorded accurately.

The control intervention is designed to simulate the procedural patient experience of a vertebroplasty. After administration of conscious sedation, local anesthetic will be injected into the subcutaneous tissues of the back in the vicinity of the fracture. A 4-mm skin incision is made and light tapping on the skin will be made to simulate vertebroplasty needle advance. Conversation regarding cement mixing and injection will be made by the operator to suggest a vertebroplasty is being performed.

### Measures

Data will be recorded at baseline, 3 days, 14 days, and 1, 3 and 6 months following procedure. During the baseline consultation patient questionnaires will be administered in a face-to-face interview. The 2-week and 6-month interviews will also be conducted in person by research staff during patient visits. The other interviews (day 3, month 1 and month 3) will be conducted by the same research staff by telephone. An exception to this will be inpatients that remain in hospital at day 3 who may be interviewed at bedside. The schedule of these data points is presented in Table 2.

**Table 2:** **Schedule for data collection**

**Evaluations**	**Visit baseline**	**Phone day 3**	**Visit day 14**	**Phone 1 month**	**Phone 3 month**	**Visit 6 month**
Procedure/operative information	X					
Pain NRS Questionnaire	X	X	X	X	X	X
Pain VAS Questionnaire	X		X			X
Physicians VAS Questionnaire	X		X			X
Roland-Morris Low Back Pain and Disability Questionnaire	X	X	X	X	X	X
Osteoporosis Quality of Life Questionnaire (QUALEFFO)	X		X	X		X
EQ-5D Questionnaire	X	X	X	X	X	X
Change in pain		X	X			
Perception of treatment assignment		X	X			
Pain medication	X	X	X	X	X	X
Timed up and go test	X		X			X
Adverse events		X	X	X	X	X
Resource use/health care utilization information						X
Erect spinal X-ray	X					X

The numeric rated scale (NRS) of pain will be used at all data collection points. Patients will be asked to answer with a number between 0 to 10 to estimate their pain intensity. This is the primary outcome measure. To standardize results, the primary pain question is the same as that used in the INVEST Trial [16], the larger of the published blinded trials of vertebroplasty. This primary pain score will record pain over the previous 24 hours. Separate numeric pain scores at rest and during standing or moving will also be obtained.

Visual analogue scale (VAS) of pain will be recorded at the three clinic visits (baseline, 2 weeks and 6 months post procedure). This score will be obtained by asking the patient to make a mark on a line which is 10 cm in length in answer to the primary pain question. The line is marked “no pain” at the left extreme and “worst possible pain” at the right extreme. There are no other divisions or marks on the line. This VAS measure is recorded for overall pain over previous 24 hours as well as separate measures for pain at rest and during walking or activity. This VAS pain score will supplement the NRS pain score at these three time points including the primary end point (NRS pain at 2 weeks post procedure). The subjective nature of patient reported pain is problematic and thus the dual pain measurements. The researcher will also record their own estimate of patient pain at the clinic visits.

The Roland-Morris Low Back Pain and Disability Questionnaire (RDQ) is a 24-item back pain specific functional status questionnaire [17]. It consists of 24 yes/no items, which represent common dysfunctions in daily activities experienced by patients with back pain. Scores range from 0 to 24, with higher numbers indicating worse physical functioning. This score will be collected at all data collection time points.

The Quality of Life Questionnaire of the European Foundation for Osteoporosis (QUALEFFO) comprises 41 quality of life questions and includes five domains: (A) pain; (B, C, D) physical function; (E) social function; (F) general health perception and (G) mental function [18]. The physical function domain is separated into three sub-domains: activities of daily living (B), jobs around the house (C) and mobility (D), so there are effectively seven categories. Each of these seven categories will be individually scored in addition to an overall score. This questionnaire is quite time consuming and lengthy for the elderly, frail patients in this trial. For this reason it will be administered at baseline, 2 weeks, one month and 6 months but not at 3 days and 3 months.

The European Quality of Life - 5 Dimensions (EQ-5D) is a health questionnaire [19]. This scale is a standardized instrument for use as a measure of health outcome. Scores range from 0 to 1, with 1 indicating perfect health. It also includes a 0 to 100 VAS to assess general health. This will be recorded at all data collection time points.

Analgesic consumption will be recorded at each time point. This will include the daily dose of each analgesic. Each medication will be classified as “strong opioid”, “mild opioid” or “non-narcotic analgesic” according to opioid comparative information published in the Australian Medicines Handbook [20]. The opioids classified as “strong” include oxycodone, morphine, fentanyl, pethidine, hydromorphone, buprenorphine and tramadol. The opioids classified as “mild” include medications containing codeine or dextropropoxyphene. The non-opioid medications include paracetamol and non-steroidal anti-inflammatory drugs (NSAIDs). The patient will be given a score of 0, 1 or 2 in each of these three analgesic medication categories depending on the number of concurrent different medications being taken within each category. If the patient is taking two different “strong” opioid preparations, for example one prolonged release and another for breakthrough pain, then the score in the strong narcotic category will be 2. If the patient is using one strong opioid medication the score in this category will be 1. If the patient is taking no “mild” narcotics the score in that category is zero. If the patient is taking either paracetamol or an NSAID, then the score in that category will be 1 and if taking both it will be 2. The scores obtained in each of these three categories will be compared between the control and vertebroplasty groups. It is our hypothesis that the “strong opioid” scores in the vertebroplasty group will be lower than those of the control group.

In addition the daily opiate dose will be converted into a morphine dose equivalent using the Opioid Dose Equivalence, Calculation of oral Morphine Equivalent Daily Dose oMEDD) [21], published by the Faculty of Pain Medicine which is a faculty of the Australian and New Zealand College of Anaesthetists. The morphine equivalent daily dose at each data point will be compared between placebo and vertebroplasty groups.

Questions regarding perceived pain change after procedure, blinding and perception of treatment assignment will be administered at 3 days and 2 weeks. A timed get up and go test [22], which is a measure of function in older people, will be performed during clinic visits at baseline, 2 weeks and 6 months. Questions regarding duration of hospitalization, resource use and health care utilization will be collected at 6 months.

### Sample size

The primary outcome is the proportion of patients achieving a 14 day pain score of less than 4 out of 10 (from a baseline score greater than or equal to 7 out of 10). A sample size of 60 patients per arm will have >80% power and 95% confidence to detect a difference in this primary outcome from 35% in the control arm to 65% in the active arm, allowing for a modest loss to follow-up. Recruitment is expected to start November 2011 and take approximately 3 years.

### Analysis

Effectiveness analyses will be by the intention-to-treat principle, whereby patients are analysed according to the study arm to which they were randomized regardless of the treatment actually received, and toxicity analyses will be according to treatment received. Proportions will be compared using a two-sided chi-squared or exact (conditional binomial) test. Changes in quality of life measures (pain and functional disability scores) will be analysed using *t*-tests to enable comparisons with published studies, and non-parametric tests or regression modelling techniques depending on the distribution of the data. All comparisons will be two-sided with a significance level of 5% considered as being statistically significant. Measures of effect will be presented as either risk differences, odds ratios or mean differences together with the respective 95% confidence intervals.

The primary end point is the proportion of patients whose NRS pain score reduces from 7/10 (or more) to 4/10 (or less) at 14 days. The study sample size is sufficient to detect an absolute difference of 30% in favour of the vertebroplasty arm. Sub-group analysis will also be performed on three different regions of the spine: thoracic (T4 to T10), thoraco-lumbar (T11 to L2) and lumbar (L3-L5). Those patients with two acute fractures involving more than one of these regions will be excluded from this sub-group analysis. We hypothesize that the vertebroplasty efficacy will be maximal in the thoracolumbar group. We expect the benefit will be greater in thoraco-lumbar fractures than in thoracic and lumbar fractures. We hypothesize that the primary end point (pain score <4/10 at 2 weeks) will be achieved in more than 30% additional patients (than control) in the thoraco-lumbar fractures and in less than 30% additional patients (than control) in the thoracic group and the lumbar group. Note that the trial is not specifically powered for this sub-group analysis, and the statistical conclusions that can be drawn from the sub-group analysis will be limited by the number of patients in each sub-group.

### Adverse events

Adverse events will be recorded during the 6 months post procedure. An adverse event is defined as any undesirable experience associated with the use of a medical product in a patient. The event is serious and should be reported within 24 hours when the patient outcome is: death, life-threatening, disability or permanent damage, or the event may jeopardize the patient and may require medical or surgical intervention (treatment) to prevent one of the other outcomes.

An independent safety committee comprising two physicians will review adverse events during the trial. As each serious adverse event (SAE) is reported, one of these physicians will review the details of the SAE within 24 hours. This physician will determine causation (was the SAE deemed related to the procedure or not) and, if related to the procedure, was the event within the adverse events provided in the information and consent.

The safety review committee will review all SAEs every 6 months to determine if each SAE was deemed to have been caused by the procedure or whether the SAE occurred (or could have occurred) as a result of comorbidities and factors other than the procedure.

### Radiologic measures

Erect radiographs of the thoracic and lumbar spine will be obtained at baseline and at 6 months in a standardized fashion to allow comparison of vertebral body heights. The heights of the fractured vertebral body in the lateral projection will be measured at the posterior margin, mid-point and anterior margin of the vertebral body. It is our hypothesis that vertebroplasty will prevent loss of vertebral body height in comparison to placebo. The X-rays will also assess for incident fractures in the two groups. Radiologic measures will be assessed by two radiologists by consensus. As it is obvious from the 6 month radiograph whether vertebroplasty has been administered, these measurements are not blinded.

### Ethics

Ethics approval has been received from the Human Research Ethics Committees of Bellberry Limited [2011-08-414] and also through the National Ethics Application Form by Northern Sydney Local Health District Human Research Ethics Committee [HREC/11/HAWKE/228]. Local approval was obtained from South Eastern Sydney local health district governance for St George Public Hospital [SSA/12/STG/30] and for Sutherland Hospital [SSA/12/STG/60]. Local approval was obtained for Liverpool Hospital from South Western Sydney local health district governance [SSA/12/LPOOL/45-12/027]. Local approval was obtained for Royal North Shore Hospital from North Sydney local health district governance [1301–009 M].

## Discussion

Although the majority of osteoporotic fractures cause minimal morbidity, there is a sub-group of patients that experience severe pain or immobility. This can be a turning point in the life of an elderly patient and can be the difference between independence and institutionalized care. This patient group may develop complications from conservative therapy. Such complications could include narcotic-induced delirium, narcotic-induced constipation, loss of confidence in mobilizing after prolonged bed rest and swelling in the lower legs due to prolonged sitting with the legs not elevated. The principle of acute intervention is to break this downward spiral and allow early mobilization, earlier rehabilitation and recovery of independence.

There are several difficulties in conducting a randomized controlled procedural trial in an elderly population with acute pain. The primary end point of this trial is the numeric rated pain score at 2 weeks. Patient-rated pain score is subjective and can be affected by other factors, particularly the use of narcotic analgesics. For this reason, the trial includes two office visits (at 2 weeks and 6 months, respectively). These visits allow for the collection of a properly performed visual analogue pain score to supplement and compare with the numeric pain score. The office visits do make the trial more difficult to conduct as elderly patients with osteoporotic fractures often have difficulty arranging transportation and require family members, often at their inconvenience, to accompany them.

The Roland Morris Disability score will provide important data as it comprises 24 yes/no questions relating to low back pain and disability. This measure is regarded by the authors as of equal importance to the pain measures. The disability caused by painful spinal fractures is of just as much clinical importance as the pain itself and the 24 yes/no questions are perhaps less subjective than the simple nomination of a pain score. Analgesic use and duration of hospitalization will also provide objective although indirect measures related to pain and disability.

The inability to provide informed consent may preclude a significant number of otherwise eligible patients from the trial. Elderly patients hospitalized with acute fracture pain are usually treated with narcotic analgesics which can induce delirium and lack of clarity in short-term memory. Patients with chronic back pain are also to be excluded. These patients may derive a clinical benefit from vertebroplasty, but this would less likely translate to a statistically significant change in pain score if the baseline pain score is chronically elevated.

Although the trial sites had existing vertebroplasty programs, we decided at all trial sites to stop offering vertebroplasty outside of the trial. As the best available evidence does not support routine use of vertebroplasty, this was an ethical decision. Patients referred for vertebroplasty now have the choice between enrolment in the trial or conservative therapy. The only means of obtaining a vertebroplasty is via enrolment in the trial. This facilitates enrolment into the trial and links the trial directly to real clinical practice.

There is no crossover in this trial. The larger of the published blinded RCTs [1] allowed crossover of patients at one month resulting in 27 of the 61 patients in the control arm crossing over to the vertebroplasty arm. The VAPOUR trial will apply otherwise similar trial methodology to the previous blinded RCTs but in a different patient group. This will provide much needed evidence regarding the clinical efficacy of vertebroplasty for painful vertebral fractures that are less than 6 weeks in duration.

## Trial status

At the time of writing (5 September 2014) 112 patients had been enrolled into the trial. At current rates of enrolment we would anticipate concluding enrolment in December 2014.

## Abbreviations

RCT: Randomized controlled trial
NHMRC: National Health and Medical Research Council (Australia)
